# Development and external validation of a model to predict recurrence in patients with non-muscle invasive bladder cancer

**DOI:** 10.3389/fimmu.2024.1467527

**Published:** 2025-01-10

**Authors:** Jiajia Tang, Longmei Fan, Tianyu Huang, Rongrong Yang, Xinqi Yang, Yuanjian Liao, Mingshun Zuo, Neng Zhang, Jiangrong Zhang

**Affiliations:** ^1^ School of Nursing, Zunyi Medical University, Zunyi, China; ^2^ Department of Urology, the Affiliated Hospital of ZunYi Medical University, Zunyi, China

**Keywords:** Lasso-Cox regression, nomogram, random forest, non-muscle invasive bladder cancer, recurrence

## Abstract

**Background:**

Most patients initially diagnosed with non-muscle invasive bladder cancer (NMIBC) still have frequent recurrence after urethral bladder tumor electrodesiccation supplemented with intravesical instillation therapy, and their risk of recurrence is difficult to predict. Risk prediction models used to predict postoperative recurrence in patients with NMIBC have limitations, such as a limited number of included cases and a lack of validation. Therefore, there is an urgent need to develop new models to compensate for the shortcomings and potentially provide evidence for predicting postoperative recurrence in NMIBC patients.

**Methods:**

Clinicopathologic characteristics and follow-up data were retrospectively collected from 556 patients with NMIBC who underwent transurethral resection of bladder tumors by electrocautery (TURBT) from January 2014 to December 2023 at the Affiliated Hospital of Zunyi Medical University and 167 patients with NMIBC who underwent the same procedure from January 2018 to April 2024 at the Third Affiliated Hospital of Zunyi Medical University. Independent risk factors affecting the recurrence of NMIBC were screened using the least absolute shrinkage and selection operator (Lasso) and Cox regression analysis. Cox risk regression models and randomized survival forest (RSF) models were developed. The optimal model was selected by comparing the area under the curve (AUC) of the working characteristics of the subjects in both and presented as a column-line graph.

**Results:**

The study included data from 566 patients obtained from the affiliated hospital of Zunyi Medical University and 167 patients obtained from the third affiliated hospital of Zunyi Medical University. Tumor number, urine leukocytes, urine occult blood, platelets, and red blood cell distribution width were confirmed as independent risk factors predicting RFS by Lasso-Cox regression analysis. The Cox proportional risk regression model and RSF model were constructed based on Lasso, which showed good predictive efficacy in both training and validation sets, especially the traditional Cox proportional risk regression model. In addition, the discrimination, consistency, and clinical utility of the column-line graph were assessed using C-index, area under the curve (AUC), calibration curve, and decision curve analysis (DCA). Patients at high risk of recurrence can be identified early based on risk stratification.

**Conclusion:**

Internal and external validation has demonstrated that the model is highly discriminative and stable and can be used to assess the risk of early recurrence in NMIBC patients and to guide clinical decision-making.

## 1 Introduction

Approximately 75% of patients with bladder cancer are diagnosed with non-muscle invasive bladder cancer (NMIBC) at their initial visi (1). Transurethral resection of bladder tumor (TURBT) supplemented with intravesical instillation is the standard treatment for NMIBC. With the rapid development of medical technology in recent years, the surgical approach has gradually matured, and the use of some novel therapeutic strategies, such as targeted therapies and immune checkpoint inhibitors, has enhanced patient outcomes. However, the prognosis of NMIBC patients still faces great challenges, mainly due to their high recurrence rate. About 50–70% of NMIBC patients will recur after surgery (2), and 10–20% of patients finally progress to muscle-invasive bladder cancer (MIBC) or metastatic bladder cancer (3). The recurrence of NMIBC is affected by a variety of factors, such as the pathological stage of the tumor, the number of tumors, the size of the tumors, and the operating level of the surgeon. Therefore, it is crucial to comprehensively analyze the risk factors affecting the recurrence of NMIBC. The main scoring models that are widely used to predict recurrence in NMIBC patients are the European Association of Urology (EAU) scoring model, the European Organization for Research and Treatment of Cancer (EORTC) scoring model, and the Spanish Urological Oncology Treatment Group (CUETO) scoring model (4–6). These models, developed for different NMIBC populations (7), cannot be fully applied to all NMIBC patients and have not yet taken into account the possible presence of biomarker variables such as blood and urine.

Previous studies have shown that changes in the values of certain inflammatory markers in the blood are associated with the recurrence of NMIBC, such as systemic immunoinflammatory index (SII) (8–11), mean platelet volume to lymphocyte ratio (12), and neutrophil/lymphocyte ratio (11, 13). Platelets are important coordinators involved in inflammatory and immune responses (14) and play an important role in key aspects of tumor cell extravasation, tumor growth, immune escape, and distant metastasis (15). Thrombocytosis has been associated with poor prognosis in a variety of cancers, such as ovarian, lung, and gastrointestinal cancers (16–18), but the predictive value of platelet count in NMIBC has not been elucidated. In addition, certain urinary markers such as albumin, globulin, and Prognostic Nutritional Index (PNI) have some predictive value for recurrence in patients with NMIBC (19). Some studies have shown that NMIBC patients with preoperative pyuria responded poorly to BCG treatment (20), and patients with pyuria had lower recurrence-free survival (RFS) and progression-free survival (PFS) than patients without pyuria (21, 22). In addition, hematuria of the naked eye (23), urine erythrocyte count (9), and urine leukocyte count may be potential predictors of RFS (24). Nonetheless, potential predictive biomarkers that may be present in urine have been rarely mentioned. These indicators obtained based on blood and urine are cost-effective, easy to obtain, and non-invasive to perform, and are expected to play an important predictive role in the prognosis of NMIBC.

Therefore, this study incorporated relevant parameters in urine and combined them with statistically significant predictors from previous studies for a comprehensive assessment. The development of a new predictive model using machine learning is dedicated to improving the predictive performance of recurrence outcomes in NMIBC patients, and external validation is used to demonstrate the accuracy and clinical applicability of the model in the real world, which will help to improve the treatment and clinical decision-making for NMIBC patients.

## 2 Methods

### 2.1 Study population

**Figure 1:**
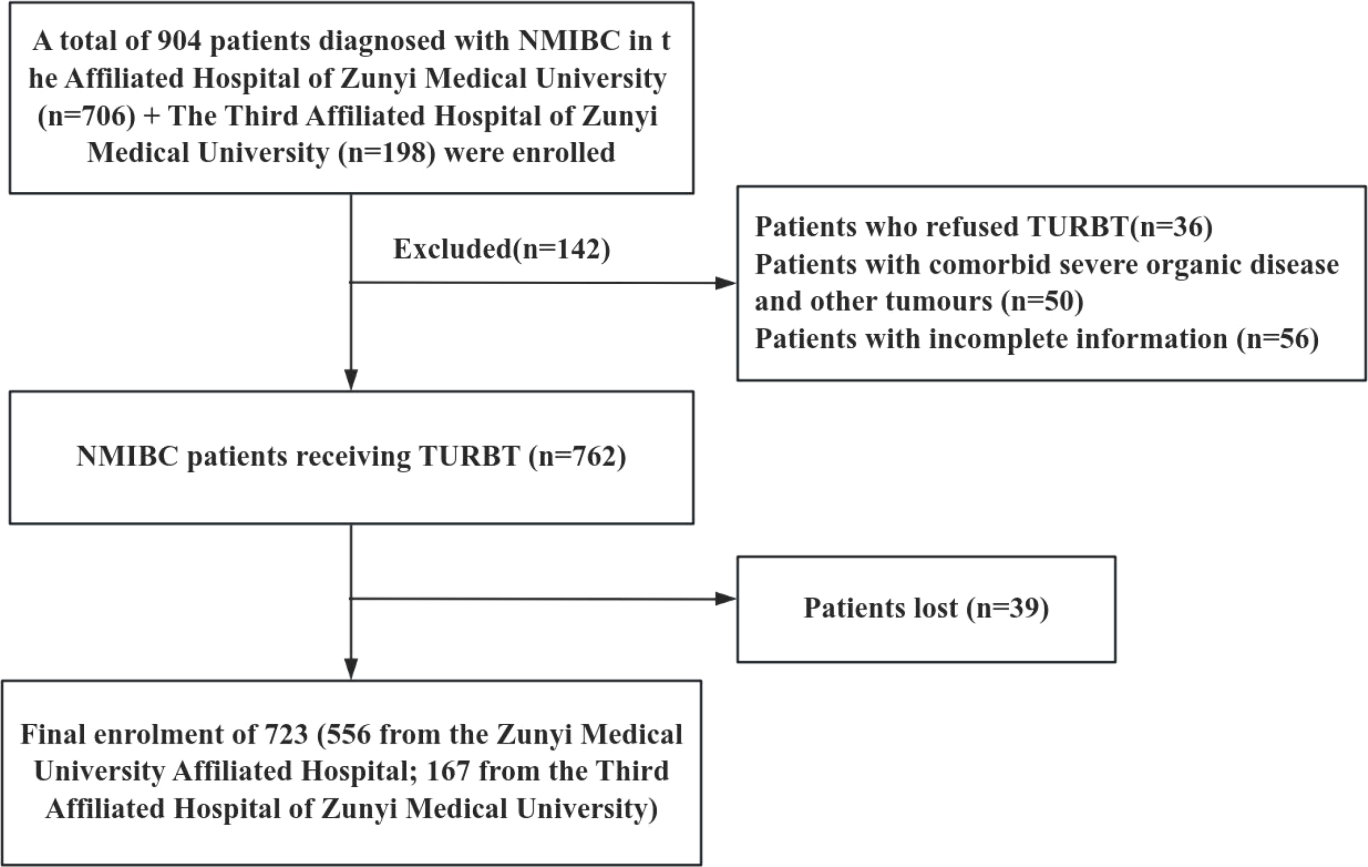
The flow diagram shows the sample selection.

Diese Studie complied with the Declaration of Helsinki and the ethical requirements of the Institutional Ethics Research Committee and was approved by the Ethics Committee of the Affiliated Hospital of Zunyi Medical University (KLLY-2023-107). A total of 556 NMIBC patients who underwent initial TURBT from January 2014 to December 2023 at the Affiliated Hospital of Zunyi Medical University were selected as an internal validation cohort. In addition, 167 NMIBC patients who underwent the same procedure at the Third Affiliated Hospital of Zunyi Medical University from January 2018 to April 2024 were selected as the external validation cohort. Inclusion criteria for the study population: (1) Patients diagnosed with NMIBC by histopathologic findings after the first TURBT; (2) Patients with complete basic clinical information and data; and (3) Patients who did not receive relevant antitumor treatments, such as radiotherapy, chemotherapy, and immunotherapy, prior to the operation. Exclusion Criteria: (1) Patients with combination of other cancers; (2) Patients with tumor recurrence, progression, or death within einem Monat nach Surgery. The case-screening process is shown in **Figure 1**.

### 2.2 Data collection

The data collected in this study included general information, laboratory findings, and clinicopathologic features of the patients. General Information: age, gender, blood type, smoking history, diabetes, hypertension, cerebrovascular disease, and urological disease; Clinicopathological Characteristics: number, size, growth location, growth mode of the tumor, bladder perfusion medication, histopathological staging and grading of the tumor; and peripheral blood laboratory test indexes in the week before the operation: blood glucose, creatinine, uric acid, albumin, globulin, total protein, A/G, platelets (PLT), hemoglobin, red blood cell distribution width (RDW), prothrombin time, and activated partial thromboplastin; and urinary laboratory indicators within 1 week before surgery: leukocytes in urine (LEU), leukocytes in urine (quantitative), urinary proteins, urinary occult blood (BLD), and urinary nitrite. NMIBC was staged according to the 8th edition of the TNM staging method, published by the International Union Against Cancer (UICC) in 2017.

### 2.3 Follow-up

Follow-up was mainly based on telephone follow-up and outpatient follow-up. Low-risk NMIBC patients underwent the 1st cystoscopy at the 3rd month postoperatively, and if the cystoscopy was negative, a second cystoscopy was performed at 1 year postoperatively, and then once a year for 5 years. Patients with high-risk NMIBC had a repeat cystoscopy every 3 months for 2 years after surgery and a repeat cystoscopy every 6 months thereafter for 5 years. The follow-up deadline was April 31, 2024. The follow-up included the drugs for bladder irrigation, the time of cystoscopy, abdominal ultrasound, and CT examination to observe postoperative tumor recurrence. If new organisms in the bladder were found on examination, a biopsy was taken under cystoscopy or tissue biopsy was taken by TURBT after admission to the hospital and sent to pathology to clarify whether the tumor was recurrent or not. Recurrence-free survival (RFS) was defined as the time to the first recurrence after surgery.

### 2.4 Statistical analysis

SPSS 29.0 software was used to statistically analyze the data. Measurement information that conformed to normal distribution was expressed as mean ± standard deviation (), and an independent samples t-test was used for comparison between groups; measurement information that did not conform to normal distribution was expressed as median (interquartile spacing), and the Mann-Whitney U test was used for comparison between groups; and counting information was described as frequency and percentile, and the χ2 test was used for comparison between groups. Survival analysis was performed using Kaplan-Meier curves using the log-rank test. The data were randomly divided into training and validation sets in a ratio of 7:3. Multifactor analysis was performed using the backward stepwise method in COX regression analysis. Lasso-Cox regression models and RSF models were constructed using R software (version 4.2.1, https://www.r-project.org). Harrell’s consistency index (C-index), area under the curve (AUC) of the subjects’ work characteristics, calibration curves, and decision curve analysis (DCA) were used to evaluate the discriminability, accuracy, and clinical applicability of the COX regression models. Specificity, sensitivity, accuracy, precision, recall, and F1 value were used to evaluate the predictive performance of the RSF model.

## 3 Results

### 3.1 Patient characteristics

A total of 556 patients were enrolled, of which 249 (44.7%) had recurrence, 199 (35.8%) were male and 50 (9.0%) were female, 68 (12.2%) were younger than 60, and 181 (32.6%) were older than or equal to 60, with a mean follow-up of 22.8 months, ranging from 1 to 60 months, and a median RFS of 22.0 months. The 1-, 3-, and 5-year cumulative recurrence rates were 21.0% (117/556), 35.0% (193/556), and 45.0% (249/556), respectively, as shown in **Supplementary Table 1**. The two groups were characterized in terms of age, number of tumors, urinary leukocytes (qualitative), urinary leukocytes (quantitative), urinary occult blood, A/G, globulin, hemoglobin, PLT, erythrocytes, RDW, and prothrombin time; activated partial thromboplastin was statistically different (P < 0.05), as shown in **Table 1**.

**Table 1:** The relationship between risk factors for recurrence and clinical pathological parameters after NMIBC electrocautery in the modeling group.

Variables	Total (n=385)	Recurrence (n=180)	Non- recurrence (n=205)	t/χ2/z	P value
Age				11.435	0.001
<60	132 (34.2)	46 (11.9)	86 (22.3)		
≥60	253 (65.7)	134 (34.8)	119 (30.9)		
Gender				0.065	0.798
Male	308 (80.0)	145 (37.7)	163 (42.3)		
Female	77 (20.0)	35 (9.1)	42 (10.9)		
Blood type				4.380	0.223
A	134 (34.8)	67 (17.4)	67 (17.4)		
B	104 (27.0)	49 (12.7)	55 (14.3)		
AB	41 (10.7)	13 (3.4)	28 (7.3)		
O	106 (27.5)	51 (13.2)	55 (14.3)		
Smoking				0.754	0.385
Yes	99 (25.7)	50 (13.0)	49 (12.7)		
No	286 (74.3)	130 (33.8)	156 (40.5)		
Diabetes mellitus				0.076	0.783
Yes	41 (10.6)	20 (5.2)	21 (5.5)		
No	344 (89.4)	160 (41.6)	184 (47.8)		
Hypertension				1.249	0.264
Yes	103 (26.8)	53 (13.8)	50 (13.0)		
No	282 (73.2)	127 (33.0)	155 (40.3)		
Cerebrovascular disease				0.008	0.930
Yes	11 (2.9)	5 (1.3)	6 (1.6)		
No	374 (97.1)	175 (45.5)	199 (51.7)		
Urinary system disease				1.262	0.261
Yes	168 (43.6)	84 (21.8)	84 (21.8)		
No	217 (56.4)	96 (24.9)	121 (31.4)		
Tumor number				128.447	<0.001
Single	248 (44.6)	45 (8.1)	203 (36.5)		
Multiple	308 (55.4)	204 (36.7)	104 (18.7)		
Tumor size				0.237	0.626
≤3cm	312 (81.0)	144 (37.4)	168 (43.6)		
>3cm	73 (19.0)	36 (9.4)	37 (9.6)		
Tumor location				4.358	0.225
Side wall	275 (71.4)	136 (35.3)	139 (36.1)		
Bladder neck	36 (9.4)	14 (3.7)	22 (5.7)		
Trigone	33(8.6)	16 (4.2)	17 (4.4)		
Other	41 (10.6)	14 (3.6)	27 (7.0)		
Growth pattern				2.252	0.813
Papilla	72 (18.7)	32 (8.3)	40 (10.4)		
Cauliflower	105 (27.3)	49 (12.7)	56 (14.5)		
Coralloid	45 (11.7)	25 (6.5)	20 (5.2)		
Carpet	14 (3.6)	5 (1.3)	9 (2.3)		
Waterweed	54 (14.0)	25 (6.5)	29 (7.5)		
Other	95 (24.7)	44 (11.4)	51 (13.2)		
Infusion medication				2.280	0.131
HydroxycamptotHecin	270 (70.1)	133 (34.5)	137 (35.6)		
Pirarubicin	115 (29.9)	47 (12.2)	68 (17.7)		
Tumor stage				0.009	0.922
Ta	155 (40.3)	72 (18.7)	83 (21.6)		
T1	230 (59.7)	108 (28.1)	122 (31.7)		
Tumor grade				3.712	0.156
Low malignant potential	112 (29.1)	50 (13.0)	62 (16.1)		
Low	203 (52.7)	90 (23.4)	113 (29.4)		
High	70 (18.2)	40 (10.4)	30 (7.8)		
LEU				136.454	<0.001
Positive	223 (40.1)	167 (30.0)	56 (10.1)		
Negative	333 (59.9)	82 (14.7)	251 (45.1)		
BLD				155.049	<0.001
Positive	114 (20.5)	110 (19.8)	4 (0.7)		
Negative	442 (79.5)	139 (25.0)	303 (54.5)		
Urinary protein				1.794	0.180
Positive	14 (3.6)	9 (2.3)	5 (1.3)		
Negative	371 (96.4)	171 (44.4)	200 (51.9)		
Urine nitrite				1.949	0.163
Positive	56 (14.5)	31 (8.1)	25 (6.5)		
Negative	329 (85.5)	149 (38.7)	180 (46.8)		
Blood sugar	-	4.9 (4.4~6.0)	4.8 (4.4~5.3)	-1.514	0.130
Creatinine	-	82.0 (73.0~94.8)	81.0 (71~91)	-0.911	0.362
Uric acid	-	346 (289~409)	340 (279.5~400.0)	-0.382	0.702
Total protein	-	65.3 ± 5.8	64.8 ± 5.6	0.752	0.452
Albumin	-	39.1 ± 4.0	39.8 ± 3.7	-1.736	0.083
Globulin	-	26.3 ± 4.3	25.0 ± 3.7	3.272	**0.001**
A/G	-	1.5 (1.3~1.7)	1.6 (1.4~1.8)	-3.301	**0.001**
Platelet	-	160.5 (114~191)	189 (161~230)	-6.630	**<0.001**
Leukocyte	-	5.9 (4.8~7.3)	5.8 (4.8~6.9)	-1.209	0.227
Erythrocyte	-	4.3 ± 0.6	4.4 ± 6.0	-2.355	**0.019**
Hemoglobin	-	133.5 (120~143)	138 (123~147)	-2.621	**0.009**
Thrombin time	-	19.5 (17.2~21.5)	17.9 (17.0~19.6)	-3.879	**<0.001**
Fibrinogen	-	2.8 (2.3~3.3)	2.7 (2.3~3.2)	-0.010	0.992
Red blood cell distribution width		13.4 (12.8~14.1)	13.1 (12.6~13.7)	2.771	**0.006**
U-LEU	-	10.0 (0.0~54.8)	5.0 (0.0~27.5)	-2.110	**0.035**
Activated partial Prothrombin	-	29.4 (25.9~34.3)	27.1 (25.0~31.9)	-3.152	**0.002**
Alanine aminotransferase	-	17.0 (12.0~24.0)	27.5 (13.0~27.5)	-1.785	0.074
Aspartate aminotransferase	-	23.0 (19.0~28.0)	24.0 (20.0~28.0)	-0.851	0.395
AST/ALT	-	1.3 (1.0~1.7)	1.3 (1.0~1.6)	-1.771	0.077

The meaning of bold values indicates P<0.05 and has statistical significance.

### 3.2 Prediction model built based on Lasso-Cox regression

**Figure 2:**
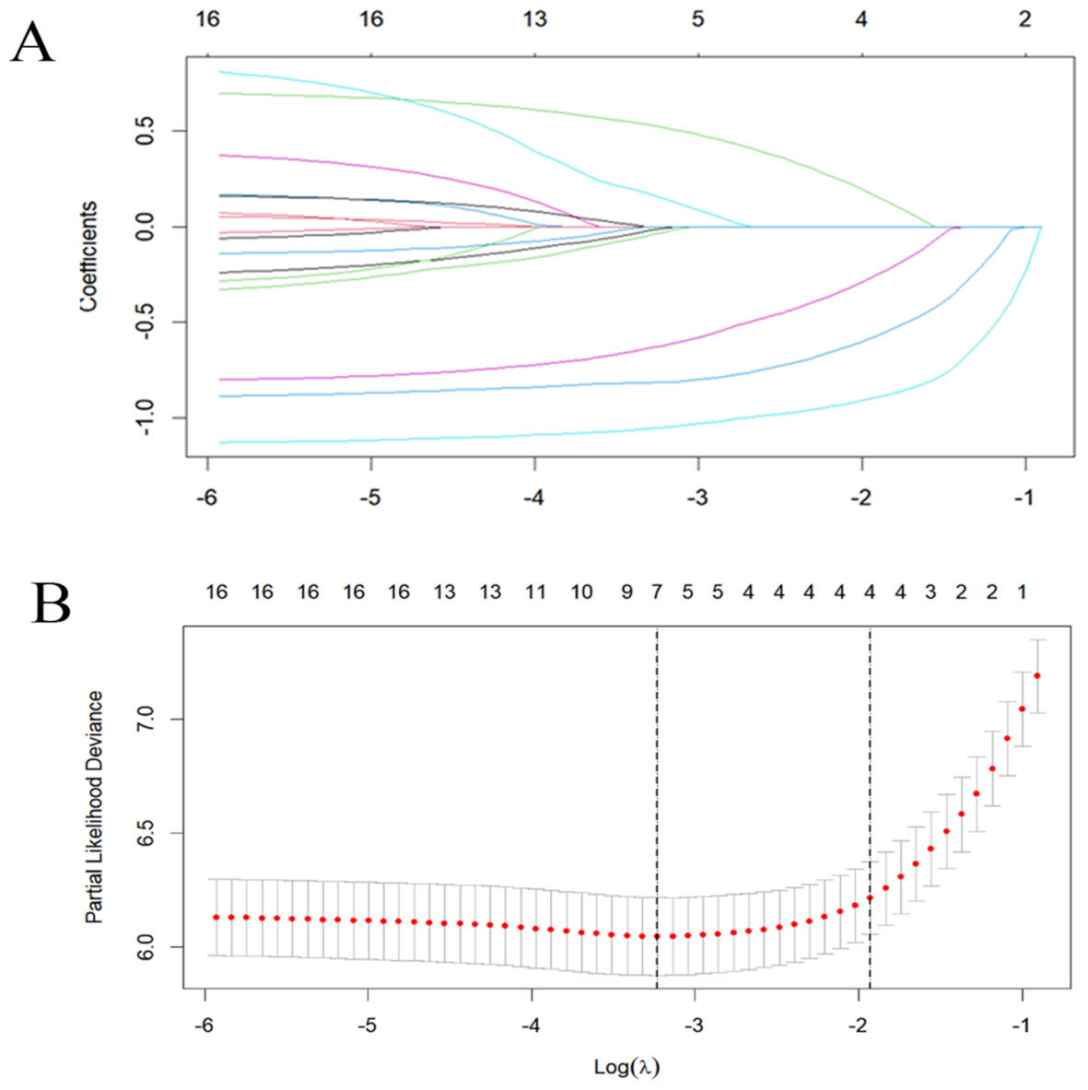
Lasso regression analysis was used to select screening variables. **(A)** The variation characteristics of variable coefficients; **(B)** The optimal λ-value process is screened by a 10-fold cross-validation process.

**Figure 3:**
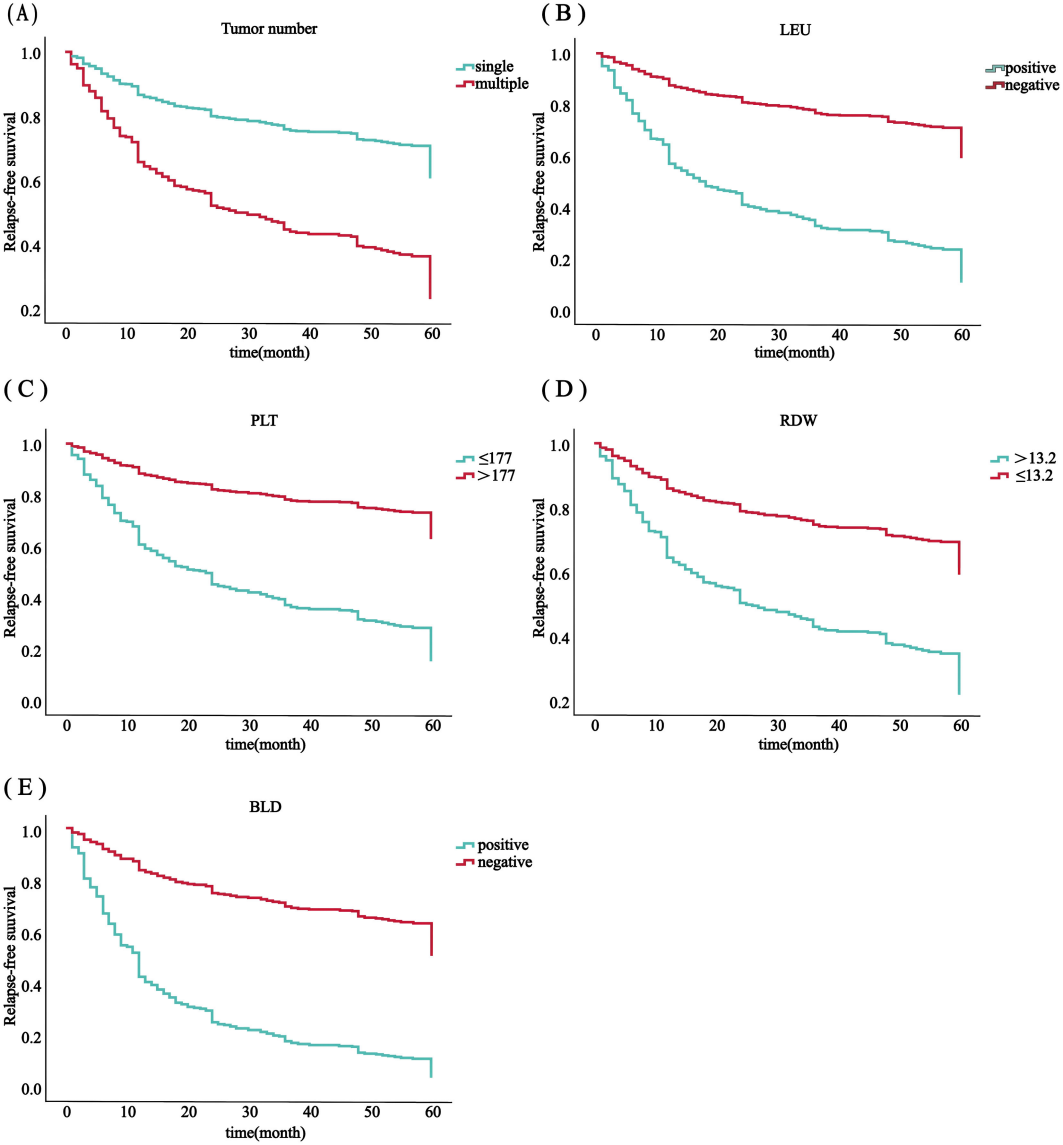
RFS analysis in all 556 patients with ovarian cancer was based on the number of tumors **(A)**, PLT **(B)**, RDW **(C)**, LEU **(D)** and BLD **(E)**.

Using SPSS software, 556 patients were randomly divided into a training set (n = 385) and an internal validation set (n = 171) in a ratio of 7:3. In order to avoid omission of potentially important factors, 16 variables with P<0.1 in the modeling set (age, tumor number, LEU, urine leukocytes (quantitative), BLD, A/G, globulin, hemoglobin, PLT, erythrocytes, RDW, prothrombin time, activated partial thromboplastin, alanine aminotransferase, AST/ALT, albumin) were screened by Lasso regression (**Figure 2**). A 10-fold cross-validation method and 52 iterations of analysis were performed using the library (GLMNET) software package, and the model with excellent performance but with the lowest number of variables was obtained at λ of 0.00352 (Log λ = 0.04342118). number of tumors: partial regression coefficient = 0.67593800, LEU: partial regression coefficient = -0.90570221, BLD: partial regression coefficient = -1.07517848, PLT: partial regression coefficient = -0.70723872, RDW: partial regression coefficient = -0.17083061 were included in Cox multifactor analysis. The results showed that BLD, LEU, RDW, and tumor number were independent risk factors for NMIBC recurrence, and PLT was a protective factor (**Table 2**). Time-dependent recurrence curves of the K-M method were plotted based on the above five indicators to see whether they predicted poor patient outcomes (**Figure 3**). A prognostic model for predicting recurrence was also constructed for screening people at high risk of recurrence for timely intervention.

**Table 2:** Cox proportional hazards regression to predict recurrence based on Lasso regression.

Variables	B	SE	Wald	HR(95.0% CI)	P value
BLD	-0.718	0.187	14.817	2.051(1.423-2.956)	0.004
PLT	-0.007	0.001	29.695	0.993(0.990-0.995)	<0.001
LEU	0.528	0.151	13.368	1.695(1.261-2.279)	<0.001
RDW	0.120	0.050	5.742	1.128(1.022-1.244)	0.017
Number of tumors	0.749	0.171	19.150	2.114(0.998-1.801)	<0.0011

### 3.3 Evaluation of the Lasso-Cox regression prediction model

**Figure 4:**
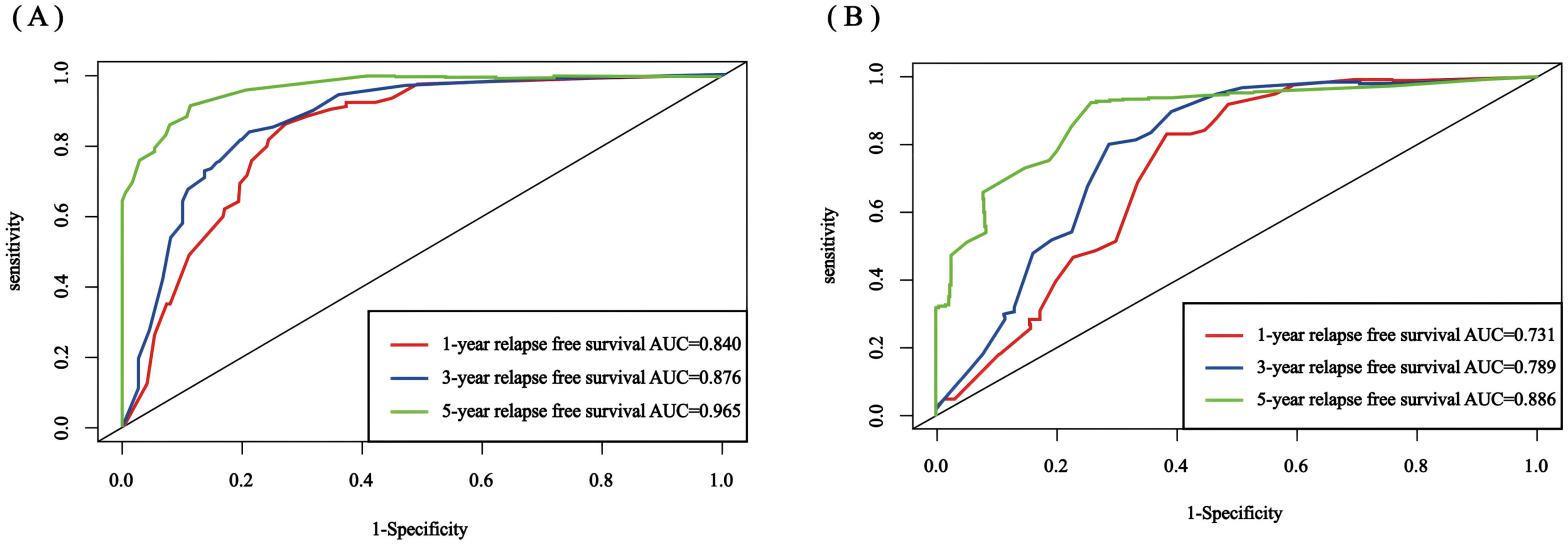
Nomogram ROC curves for 1-, 3-, and 5-year RFS for the training set and internal validation set. **(A)** training set; **(B)** internal validation set. ROC, receiver operating characteristic; AUC, area under the curve; RFS, recurrence-free survival.

**Figure 5:**
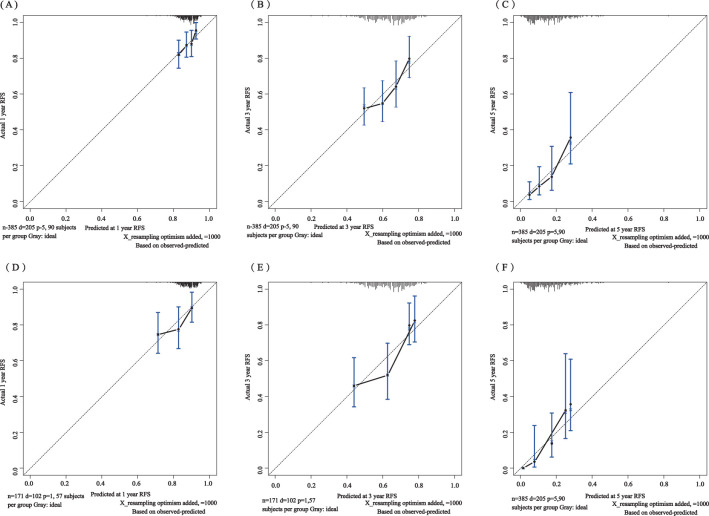
Calibration curves of the training set for 1-year **(A)**, 3-year **(B)**, and 5-year **(C)** and the internal validation set for 1-year **(D)**, 3-year **(E)**, and 5-year **(F)**. The blue curve is the prediction curve, and the dashed line is the reference curve.

**Figure 6:**
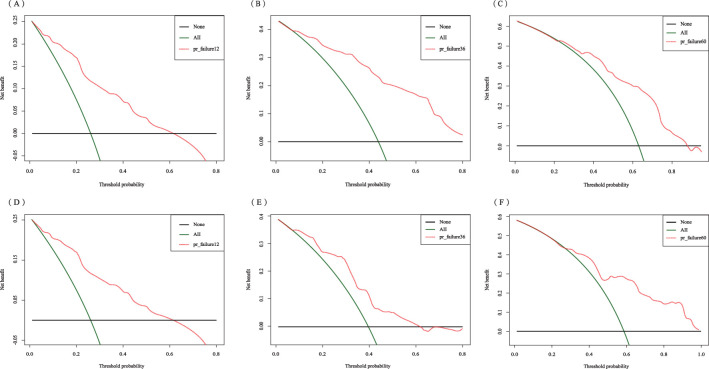
Decision curve analysis of the training set for 1-year **(A)**, 3-year **(B)**, and 5-year **(C)** and the internal validation set for 1-year **(D)**, 3-year **(E)**, and 5-year **(F)**.

The C-index for the training set and internal validation set of the COx regression model was calculated to be 0.821 (95% CI: 0.792~0.850) and 0.726 (95% CI: 0.669~0.783), respectively, by calling the library(survival) package. The model was trained by a 1000-time resampling method through a library (PEC) software package. The area under the ROC curve AUCs of the RFS for 1, 3, and 5 years in the training and internal validation sets were plotted using the library (survival ROC) and library (foreign) software packages were 0.840, 0.876, and 0.965; and 0.731, 0.789, and 0.886, respectively (**Figures 4A, B**). The results showed that the model exhibited high accuracy in predicting recurrence in patients within 5 years. The calibration curves and clinical decision curves for RFS at 1, 3, and 5 years in the training and internal validation sets were plotted using the library(rms) and library(survival) software packages, and the predicted probabilities were closer to the actual direction, indicating a high consistency of the model (**Figures 5A–F**). In addition, we also assessed the clinical applicability of the model, and DCA demonstrated that patients achieved better net clinical benefit (**Figures 6A–F**).

### 3.4 Prediction model built based on a random survival forest

**Figure 7:**
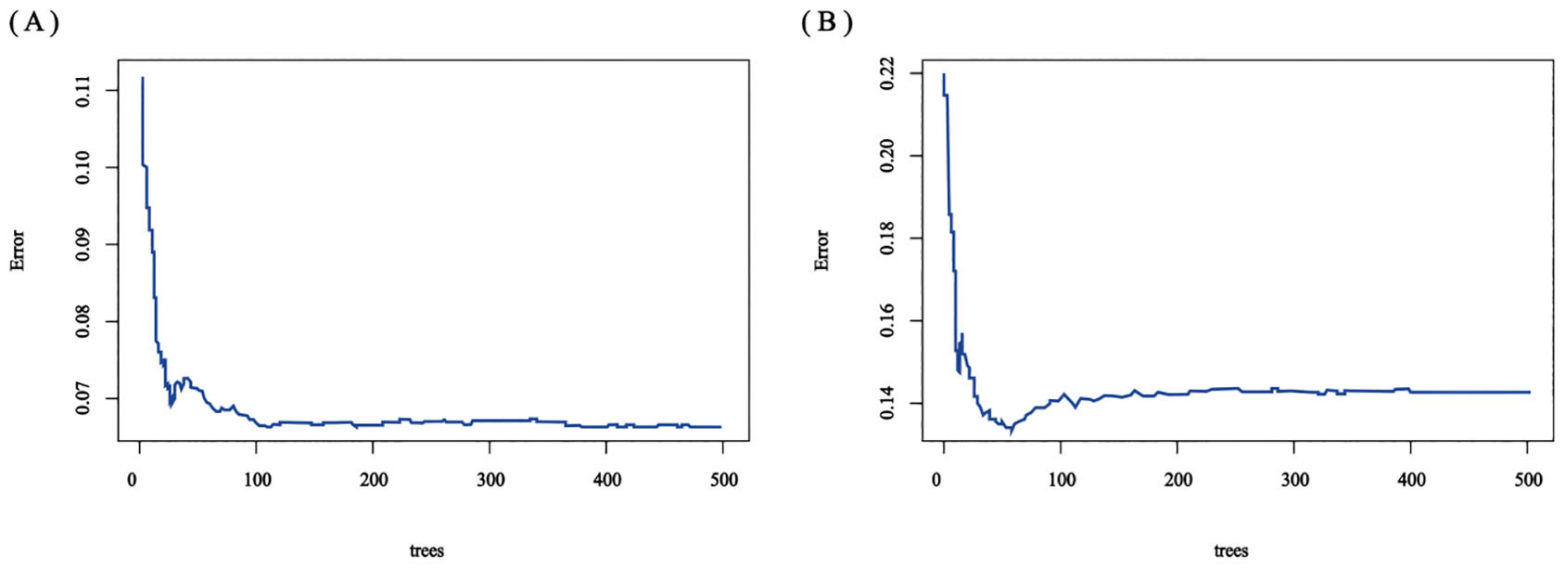
The error rate of a random survival forest. **(A)** training set; **(B)** validation set.

**Figure 8:**
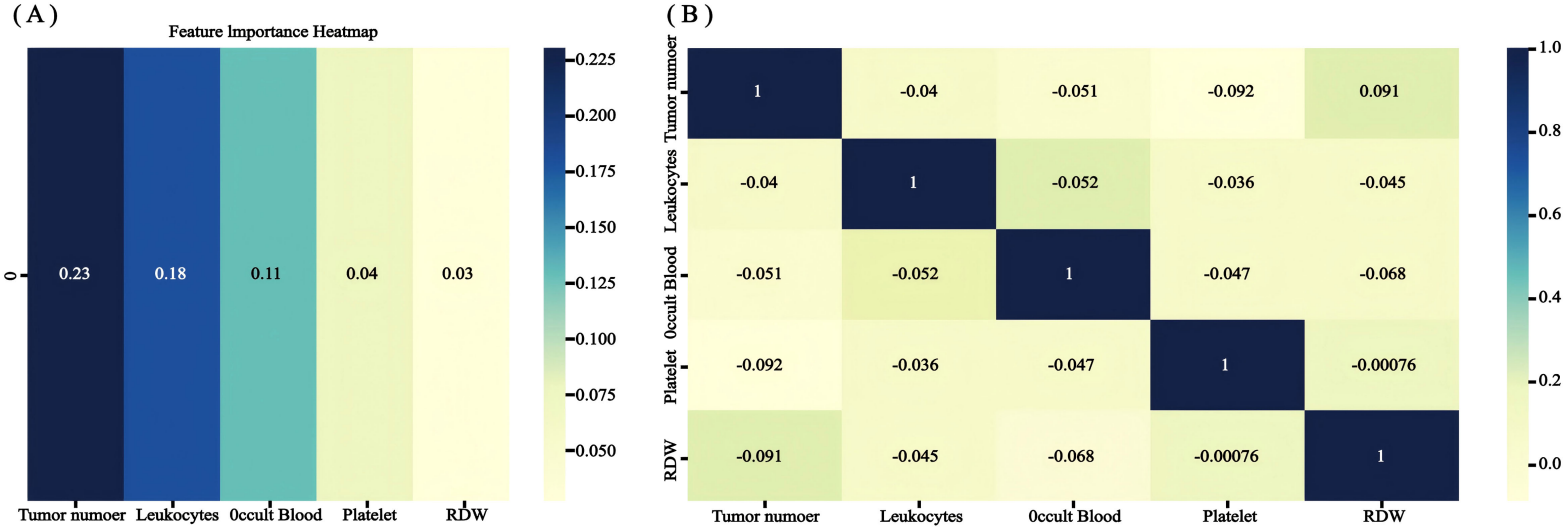
The recurrence risk analysis of NMIBC is based on a random survival forest. **(A)** Out-of-bag variable importance ranking. **(B)** correlation heat map.

The RSF model was constructed using the randomForest, caret, and pROC software packages. When setting the random seed to 123 and adjusting the parameter Mtry = 2, the training set ntree was 400, Out of bag (OOB) = 8.83%, and the squared mean of residuals (MSE) = 0.07; the validation set ntree was 250, OOB = 18.34%, and the MSE = 0.14, and the error rate of the model was stabilized (**Figures 7**). The importance of the variables was ranked according to the VIMP method, and they were, in order, tumor number, LEU, BLD, PLT, and RDW (**Figure 8**). Among them, tumor number had the strongest importance, and RDW had the weakest importance.

### 3.5 Evaluation of random forest prediction models

**Figure 9:**
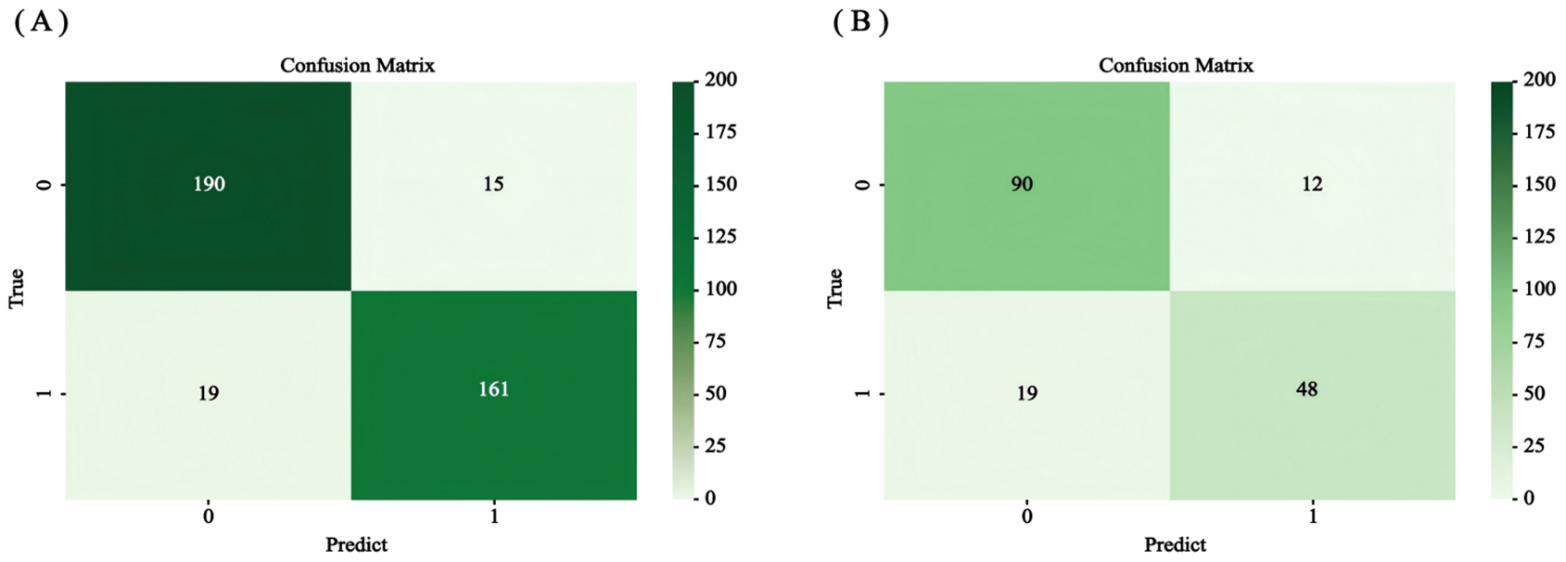
The confusion matrix is **(A)** the training set and **(B)** the validation set.

**Figure 10:**
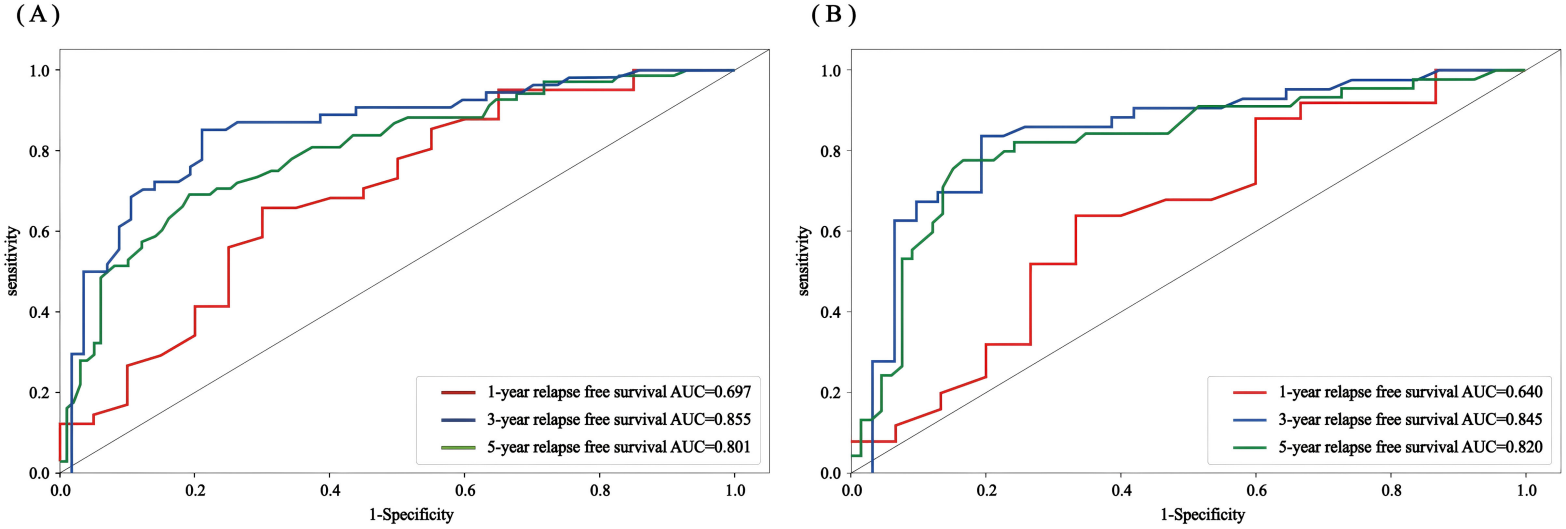
Random forest ROC curves of the 1-, 3-, and 5-year RFS for the training set and validation set **(A)** training set; **(B)** validation set.

The accuracy, precision, recall, F1 value, sensitivity, and specificity values of the RSF model were calculated by calling the caret software package to draw the confusion matrix (**Figure 9**) to assess the accuracy and generalization ability of the RSF model (**Supplementary Table 2**). The results show that the model exhibits good prediction and generalization ability in both the training and validation sets. In addition, the AUC values (0.697, 0.855, and 0.801; 0.640, 0.845, and 0.820, respectively) at 1, 3, and 5 years for the training and validation sets indicated that the model had good accuracy in predicting the RFS of the patients at 1, 3, and 5 years after the operation (**Figure 10**).

### 3.6 Nomogram as a tool for visualization

**Figure 11:**
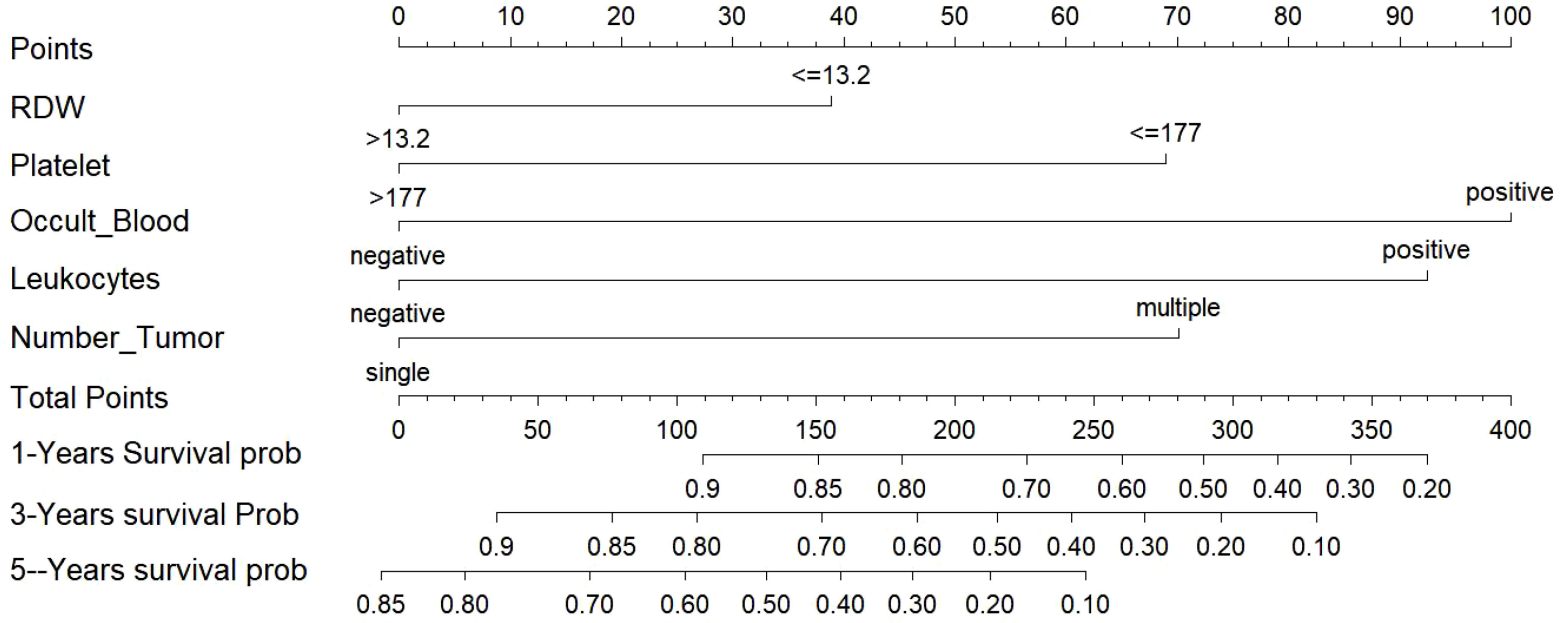
Nomogram for predicting NMIBC patients at 1-, 3-, and 5-years. NMIBC: non-muscle-invasive bladder cancer.

The empirical validation found that both models showed good predictive efficacy, so we finally chose the Lasso-COX risk-proportionality-based model as the optimal model by comparing the AUC values of the 2 models and visualizing the model in the form of a column-line graph (**Figure 11**). When using the model to assess individual risk, each variable score in the model needs to be summed. A vertical line is first drawn at the total score so that it intersects the three lines representing the predicted RFS. The values corresponding to the intersection points are the individual’s predicted 1-, 3-, and 5-year RFS. For example, an NMIBC patient with multiple tumors, positive urinary occult blood, negative urinary leukocytes, platelets of 177 × 10^9/L, and erythrocyte distribution width of >13.2% would have a total score of approximately 238, a 1-year RFS of approximately 67%, a 3-year RFS of approximately 42%, and a 5-year RFS of approximately 13%. It can be seen that a nomogram can intuitively assess the weight of each variable and help clinicians make better diagnostic and therapeutic decisions to better serve the clinic.

### 3.7 External validation of Lasso Cox regression prediction model

**Figure 12:**
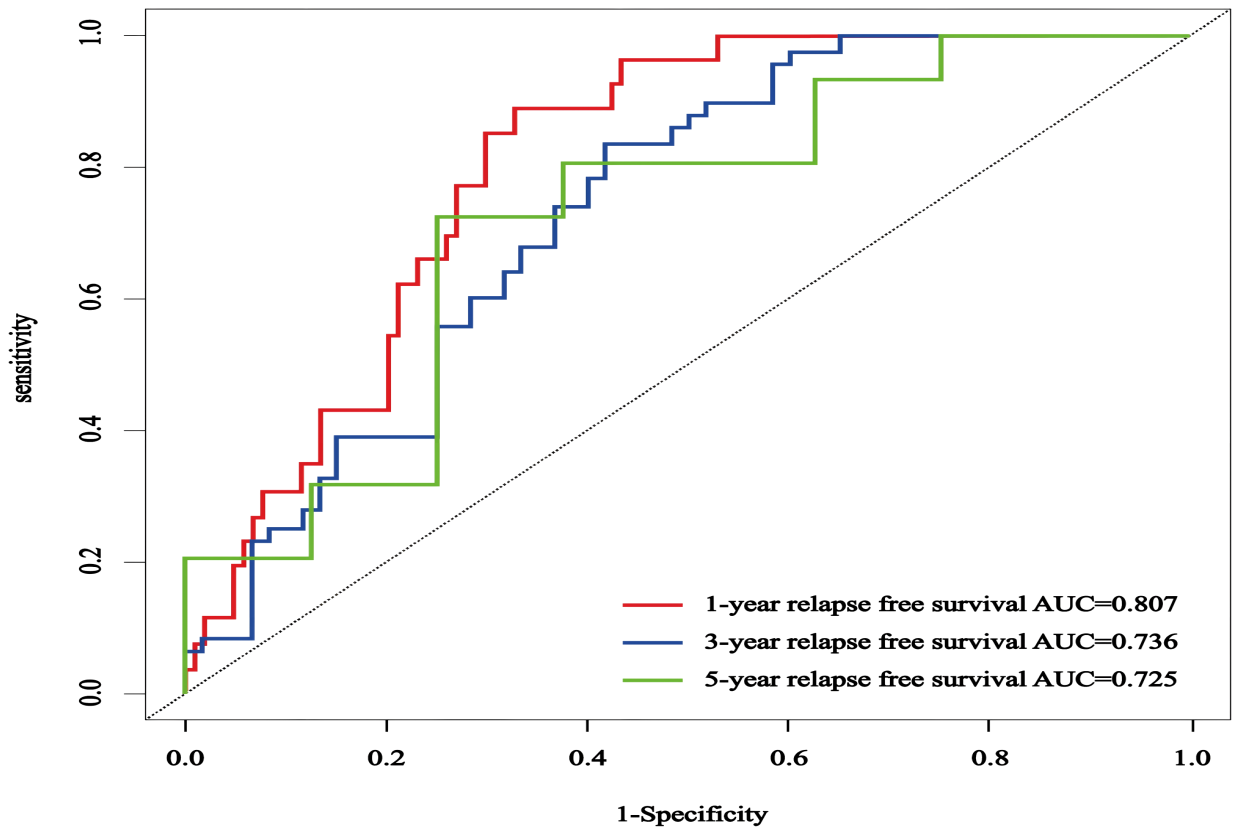
The ROC curves of an external validation cohort were employed to forecast the 1-, 3-, and 5-year rates of RFS in patients with NMIBC.

**Figure 13:**
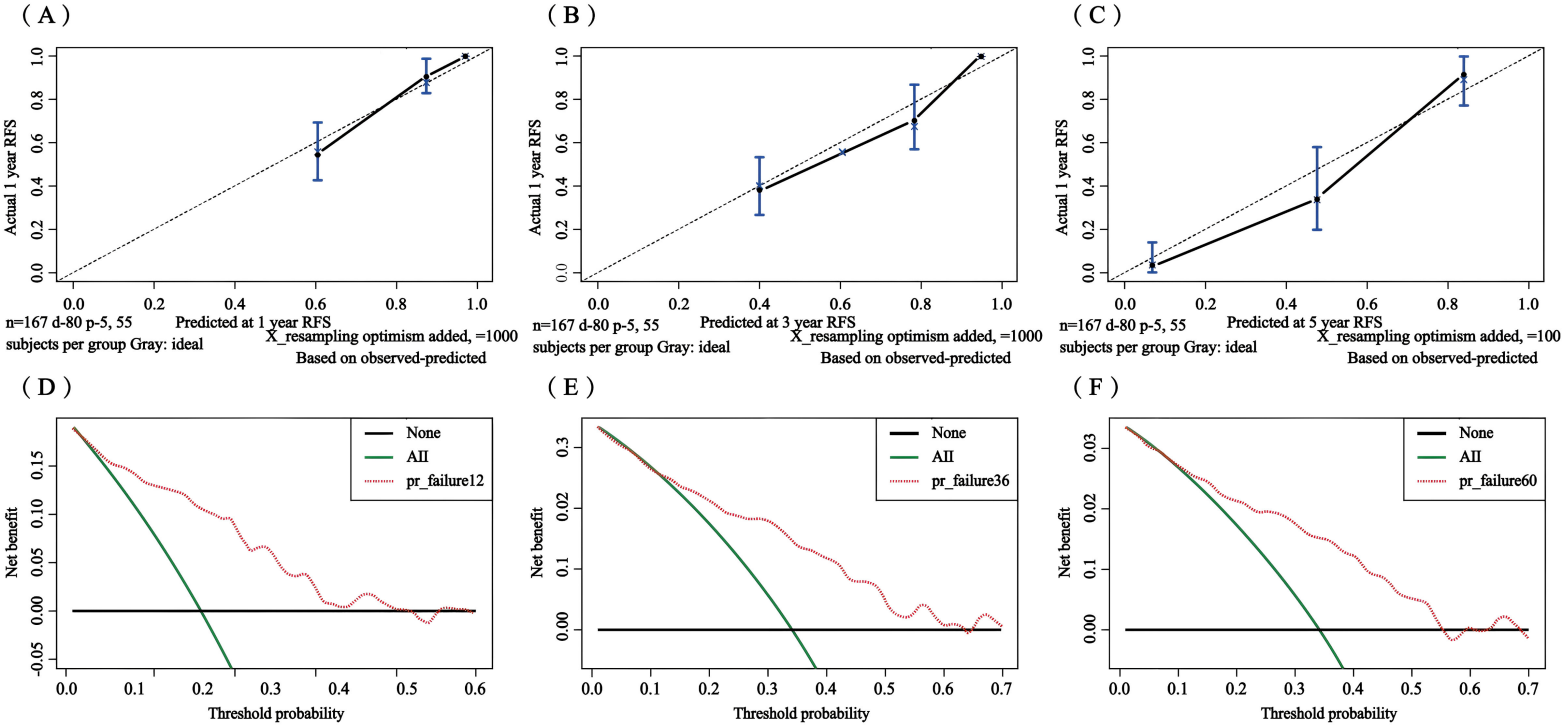
Calibration curves and clinical decision curves at 1, 3, and 5 years for the external validation set. Calibration curves: 1-year **(A)**, 3-year **(B)**, and 5-year **(C)**; clinical decision curves: 1-year **(D)**, 3-year **(E)**, and 5-year **(F)**.

The C-index in the external validation cohort was 0.776 (95% CI: 0.721-0.830). The 1-, 3-, and 5-year AUC values for patients were 0.807, 0.736, and 0.725, respectively (**Figure 12**), indicating good predictive performance of the RSF model. The calibration curves showed that the predicted probabilities in the external validation cohort were close to the same direction as the actual results (**Figures 13A–C**). The DCA confirmed the clinical validity of our column-line graphs for RFS **Figures 13D–F**), which shows that our model yielded a better net clinical benefit. In summary, the column-line diagram we developed has better predictive performance.

## 4 Discussion

The high recurrence rate is one of the main reasons plaguing the treatment of NMIBC. The postoperative recurrence rate of NMIBC in our study was 44.7%, which is within the reported range of 31-78% (25). Therefore, early identification of predisposing factors for recurrence is crucial for the treatment of NMIBC.

The number of tumors as an independent risk factor for recurrence in NMIBC is not controversial and will not be discussed here (26, 27). Elevated PLT counts have been reported to be positively correlated with more advanced stages of solid tumors, such as ovarian and lung cancer (28, 29). In hepatocellular carcinoma, patients with PLT counts ≥148 × 10^9^/L have a higher recurrence rate (30). PLT is also an independent risk factor for lymph node metastasis in MIBC (31). However, in our study, PLT was a protective rather than a risk factor for NMIBC recurrence, and patients with PLT ≤152×10^9/L had a shorter RFS than patients with >152×10^9/L. This suggests that reduced PLT is associated with a poor prognosis in BCa, but a large number of studies are needed to confirm our view.

RDW is an important parameter corresponding to the degree of homogeneity of erythrocyte volume and is one of the potential inflammatory markers to predict the prognosis of various cancers. Some studies have shown that elevated RDW is significantly and positively correlated with endometrial cancer stage, lymph node metastasis and recurrence (32). In addition, elevated RDW has been associated with lung cancer (33) and metastatic renal cell carcinoma (34), which are associated with a poorer prognosis and with ICU mortality in critically ill cancer patients (35). However, the relationship between RDW and BCa remains controversial. One study found that RDW was not associated with the recurrence of NMIBC (36, 37), which contradicts the study by Fukuoka et al (38). In the present study, we showed that the risk of BCa recurrence in the high RDW group was approximately twice that of the low RDW group, and patients with RDW > 13.2% had a shorter RFS, which is consistent with the study by Fukuoka et al.

Due to the strong association between urine and bladder tumors, urine-based diagnostic biomarkers (e.g. urine pH, urine protein, urine glucose, and BLD) have been developed for the diagnosis and prognosis of BCa. Lower urine pH and higher urine protein, urine glucose, and urinary occult blood levels were found to be strongly associated with an increased risk of BCa in a case-control study (39). Our results showed that BLD positivity was strongly associated with recurrence in patients with NMIBC, and the positive group had a shorter RFS compared to the BLD-negative group. Hematuria is the most common symptom of BCa, and although there is no direct correlation between hematuria and the extent of the disease, our results suggest a relationship between urinary parameters and the biological behavior of the tumor. Further cystoscopy is recommended for patients presenting with symptoms of BCa, particularly those with recurrent hemorrhagia or recurrent disease.

Tumor-inflammation interactions offer new immunotherapeutic avenues for cancer treatment (40). Inflammatory factors in serum and urine have also been widely used to predict the prognosis of various tumors, including BCa (41, 42). Qu (43) et al. analyzed peripheral blood leukocytes and 112 urine samples from 100 patients with NMIBC and found that interleukin-8 (IL-8) levels were high in both blood and urine in patients with recurrence. Patients with high blood IL-8 levels had a more than 2.5-fold increased risk of recurrence, and IL-8 predicted a shorter RFS, while patients with high urinary IL-8 levels had an almost 4-fold increased risk of tumor recurrence, and their RFS was similar to that of patients with low urinary IL-8 levels. This suggests that elevated levels of IL-8, both in blood and urine, are associated with BCa recurrence. In our study, we found that LEU was associated with BCa recurrence, and the positive group had a 1.7-fold higher risk of BCa recurrence and shorter RFS compared to the negative LEU group, suggesting that LEU may be a promising biomarker for predicting recurrence in NMIBC patients.

However, our research has certain limitations. Firstly, due to the retrospective nature of the study, there may be selection bias in data collection. Secondly, the data collection time span is too long, and due to the continuous updates of detection technology, there may be deviations in the test results. In addition, we only collected preoperative test results from patients and did not observe any changes in them after surgery. Finally, more clinical trials and prospective studies are needed to further demonstrate the clinical value of my research findings for the prognosis of NMIBC patients.

## 5 Conclusion

In this study, the traditional Cox proportional hazards model and the RSF model based on Lasso regression were constructed, and both models showed good predictive performance. Since the RSF model was inferior to the traditional linear regression model in predicting the recurrence rate of patients at 1 year, the Lasso-Cox model was finally selected as the optimal model, and a column chart was constructed to predict the recurrence of patients with early NMIBC. The column chart has a significant risk stratification effect and is a useful guide for screening people at high risk of recurrence.

## Data Availability

The original contributions presented in the study are included in the article/**Supplementary Material.** Further inquiries can be directed to the corresponding author.

## References

[1] Babjuk M Burger M Capoun O Cohen D Compérat EM Dominguez Escrig JL. European association of urology guidelines on non-muscle-invasive bladder cancer (Ta, T1, and carcinoma in situ). Eur Urol. (2022) 81:75–94. doi: 10.1016/j.eururo.2021.08.010 34511303

[2] Knowles MA Hurst CD. Molecular biology of bladder cancer: new insights into pathogenesis and clinical diversity. Nat Rev Cancer. (2015) 15:25–41. doi: 10.1038/nrc3817 25533674

[3] Patel VG Oh WK Galsky MD. Treatment of muscle-invasive and advanced bladder cancer in 2020. CA Cancer J Clin. (2020) 70:404–23. doi: 10.3322/caac.21631 32767764

[4] Sylvester RJ van der Meijden AP Oosterlinck W Witjes JA Bouffioux C Denis L. Predicting recurrence and progression in individual patients with stage Ta T1 bladder cancer using EORTC risk tables: a combined analysis of 2596 patients from seven EORTC trials. Eur Urol. (2006) 49:466–5. doi: 10.1016/j.eururo.2005.12.031 16442208

[5] Fernandez-Gomez J Madero R Solsona E Unda M Martinez-Piñeiro L Gonzalez M. Predicting nonmuscle invasive bladder cancer recurrence and progression in patients treated with bacillus Calmette-Guerin: the CUETO scoring model. J Urol. (2009) 182:2195–203. doi: 10.1016/j.juro.2009.07.016 19758621

[6] Sylvester RJ Rodríguez O Hernández V Turturica D Bauerová L Bruins HM. European association of urology (EAU) prognostic factor risk groups for non-muscle-invasive bladder cancer (NMIBC) incorporating the WHO 2004/2016 and WHO 1973 classification systems for grade: an update from the EAU NMIBC guidelines panel. Eur Urol. (2021) 79:480–8. doi: 10.1016/j.eururo.2020.12.033 33419683

[7] Jobczyk M Stawiski K Fendler W Różański W. Validation of EORTC, CUETO, and EAU risk stratification in prediction of recurrence, progression, and death of patients with initially non-muscle-invasive bladder cancer (NMIBC): A cohort analysis. Cancer Med. (2020) 9:4014–25. doi: 10.1002/cam4.v9.11 PMC7286464 32216043

[8] Ke ZB Chen H Chen JY Cai H Lin YZ Sun XL. Preoperative abdominal fat distribution and systemic immune inflammation were associated with response to intravesical Bacillus Calmette-Guerin immunotherapy in patients with non-muscle invasive bladder cancer. Clin Nutr. (2021) 40:5792–801. doi: 10.1016/j.clnu.2021.10.019 34775222

[9] Ding L Deng X Xia W Wang K Zhang Y Zhang Y. Development and external validation of a novel nomogram model for predicting postoperative recurrence-free survival in non-muscle-invasive bladder cancer. Front Immunol. (2022) 13:1070043. doi: 10.3389/fimmu.2022.1070043 36458001 PMC9706099

[10] Liu P Chen S Gao X Liang H Sun D Shi B. Preoperative sarcopenia and systemic immune-inflammation index can predict response to intravesical Bacillus Calmette-Guerin instillation in patients with non-muscle invasive bladder cancer. Front Immunol. (2022) 13:1032907. doi: 10.3389/fimmu.2022.1032907 36225922 PMC9549861

[11] Chakra MA Lassila R El Beayni N Mott SL O'Donnell MA. Prognostic role of the neutrophil/lymphocyte ratio in high-risk BCG-naïve non-muscle-invasive bladder cancer treated with intravesical gemcitabine/docetaxel. BJU Int. (2024) 135:125–32. doi: 10.1111/bju.16486 39082304

[12] Wang C Jin W Ma X Dong Z. The different predictive value of mean platelet volume-to-lymphocyte ratio for postoperative recurrence between non-muscular invasive bladder cancer patients treated with intravesical chemotherapy and intravesical chemohyperthermia. Front Oncol. (2022) 12:1101830. doi: 10.3389/fonc.2022.1101830 36713575 PMC9874935

[13] D'andrea D Moschini M Gust K Abufaraj M Özsoy M Mathieu R. Prognostic role of neutrophil-to-lymphocyte ratio in primary non-muscle-invasive bladder cancer. Clin Genitourin Cancer. (2017) 15:e755–64. doi: 10.1016/j.clgc.2017.03.007 28420564

[14] Thomas MR Storey RF. The role of platelets in inflammation. Thromb Haemost. (2015) 114:449–58. doi: 10.1160/TH14-12-1067 26293514

[15] Schlesinger M. Role of platelets and platelet receptors in cancer metastasis. J Hematol Oncol. (2018) 11:125. doi: 10.1186/s13045-018-0669-2 30305116 PMC6180572

[16] Said SA Inthout J Den Ouden JE Walraven JEW van der Aa MA de Hullu JA. Development and internal validation of nomograms for survival of advanced epithelial ovarian cancer based on established prognostic factors and hematologic parameters. J Clin Med. (2024) 13:2789. doi: 10.3390/jcm13102789 38792332 PMC11122536

[17] Anderson R Rapoport BL Steel HC Theron AJ. Pro-tumorigenic and thrombotic activities of platelets in lung cancer. Int J Mol Sci. (2023) 24:11927. doi: 10.3390/ijms241511927 37569299 PMC10418868

[18] Faria AVS Yu B Mommersteeg M de Souza-Oliveira PF Andrade SS Spaander MCW. Platelet-dependent signaling and Low Molecular Weight Protein Tyrosine Phosphatase expression promote aggressive phenotypic changes in gastrointestinal cancer cells. Biochim Biophys Acta Mol Basis Dis. (2022) 1868:166280. doi: 10.1016/j.bbadis.2021.166280 34610471

[19] Ye J Tang C Wu R Tang Y Yin H Bai Y. Preoperative blood-based nutritional biomarkers as significant prognostic factors after intravesical BCG therapy in patients with non-muscle-invasive bladder cancer. World J Urol. (2024) 42:428. doi: 10.1007/s00345-024-05148-1 39037600

[20] Suh J Yuk HD Jeong CW Kwak C Kim HH Ku JH. Pyuria as a predictive marker of bacillus calmette-guérin unresponsiveness in non-muscle invasive bladder cancer. J Clin Med. (2021) 10:3764. doi: 10.3390/jcm10173764 34501211 PMC8432248

[21] Azuma T Nagase Y Oshi M. Pyuria predicts poor prognosis in patients with non-muscle-invasive bladder cancer. Clin Genitourin Cancer. (2013) 11:331–6. doi: 10.1016/j.clgc.2013.04.002 23664207

[22] Satake N Ohno Y Nakashima J Ohori M Tachibana M. Prognostic value of preoperative pyuria in patients with non-muscle-invasive bladder cancer. Int J Urol. (2015) 22:645–9. doi: 10.1111/iju.2015.22.issue-7 25912166

[23] Kim HS Jeong CW Kwak C Kim HH Ku JH. Novel nomograms to predict recurrence and progression in primary non-muscle-invasive bladder cancer: validation of predictive efficacy in comparison with European Organization of Research and Treatment of Cancer scoring system. World J Urol. (2019) 37:1867–77. doi: 10.1007/s00345-018-2581-3 30535715

[24] He Y Pan C Zhang Y Lv M Yang B. Nomogram for customized recurrence prediction in primary non-muscle-invasive bladder cancer based on routine blood and urine parameters. BMC Urol. (2024) 24:67. doi: 10.1186/s12894-024-01437-4 38528549 PMC10964628

[25] Lopez-Beltran A Cookson MS Guercio BJ Cheng L. Advances in diagnosis and treatment of bladder cancer. BMJ. (2024) 384:e076743. doi: 10.1136/bmj-2023-076743 38346808

[26] Shen Z Xie L Chen T Tian D Liu X Xu H. Risk factors predictive of recurrence and progression for patients who suffered initial recurrence after transurethral resection of stage pT1 bladder tumor in chinese population: A retrospective study. Med (Baltimore). (2016) 95:e2625. doi: 10.1097/MD.0000000000002625 PMC4748891 26844474

[27] Van Rhijn BWG Burger M Lotan Y Solsona E Stief CG Sylvester RJ. Recurrence and progression of disease in non-muscle-invasive bladder cancer: from epidemiology to treatment strategy. Eur Urol. (2009) 56:430–42. doi: 10.1016/j.eururo.2009.06.028 19576682

[28] Abdulrahman GO Das N Lutchman Singh K. The predictive role of thrombocytosis in benign, borderline and Malignant ovarian tumors. Platelets. (2020) 31:795–800. doi: 10.1080/09537104.2019.1686755 31665945

[29] Skorek P Stępień K Fila M Hauer J Kużdżał J. Preoperative thrombocytosis in surgically treated patients with non-small cell lung cancer. Pol Arch Intern Med. (2018) 128:512–7. doi: 10.20452/pamw.4299 30057382

[30] Pang Q Zhang J-Y Xu X-S Song SD Qu K Chen W. Significance of platelet count and platelet-based models for hepatocellular carcinoma recurrence. World J Gastroenterol. (2015) 21:5607–21. doi: 10.3748/wjg.v21.i18.5607 PMC4427685 25987786

[31] Qiao Y Jia Y Luo L Li B Xie F Wang H. Development and validation of a nomogram to predict lymph node metastasis in patients with progressive muscle-invasive bladder cancer. Front Oncol. (2024) 14:1342244. doi: 10.3389/fonc.2024.1342244 38817904 PMC11137274

[32] Eoh K-J Lee T-K Nam E-J Kim SW Kim YT. Clinical relevance of red blood cell distribution width (RDW) in endometrial cancer: A retrospective single-center experience from korea. Cancers (Basel). (2023) 15:3984. doi: 10.3390/cancers15153984 37568799 PMC10417026

[33] Wang Y Zhou Y Zhou K Li J Che G. Prognostic value of pre-treatment red blood cell distribution width in lung cancer: a meta-analysis. Biomarkers. (2020) 25:241–7. doi: 10.1080/1354750X.2020.1731763 32064949

[34] Wei Z Zhang F Ma X He W Gou X Zhang X. Preoperative red blood cell distribution width as an independent prognostic factor in metastatic renal cell carcinoma. Transl Oncol. (2022) 23:101486. doi: 10.1016/j.tranon.2022.101486 35839619 PMC9287633

[35] Zhou J Feng L Zhang Y Huang S Jiang H Wang W. High red blood cell distribution width is associated with the mortality of critically ill cancer patients: A propensity-matching study. Adv Clin Exp Med. (2023) 32:31–41. doi: 10.17219/acem/152635 36226693

[36] Yildiz HA Değer MD Aslan G. Prognostic value of preoperative inflammation markers in non-muscle invasive bladder cancer. Int J Clin Pract. (2021) 75:e14118. doi: 10.1111/ijcp.14118 33636055

[37] Ma W Mao S Bao M Wu Y Guo Y Liu J. Prognostic significance of red cell distribution width in bladder cancer. Transl Androl Urol. (2020) 9:295–302. doi: 10.21037/tau.2020.03.08 32420135 PMC7215002

[38] Fukuokaya W Kimura T Miki J Kimura S Watanabe H Bo F. Red cell distribution width predicts time to recurrence in patients with primary non-muscle-invasive bladder cancer and improves the accuracy of the EORTC scoring system. Urol Oncol. (2020) 38:638.e15–638.e23. doi: 10.1016/j.urolonc.2020.01.016 32184059

[39] Wang D-Q Shuai J Zheng H Guo ZQ Huang Q Xu XF. Can routine blood and urine parameters reveal clues to detect bladder cancer? A case-control study. Front Oncol. (2021) 11:796975. doi: 10.3389/fonc.2021.796975 35127507 PMC8813745

[40] Klapp V Álvarez-Abril B Leuzzi G Kroemer G Ciccia A Galluzzi L. The DNA damage response and inflammation in cancer. Cancer Discovery. (2023) 13:1521–45. doi: 10.1158/2159-8290.CD-22-1220 37026695

[41] Masson-Lecomte A Rava M Real FX Hartmann A Allory Y Malats N. Inflammatory biomarkers and bladder cancer prognosis: a systematic review. Eur Urol. (2014) 66:1078–91. doi: 10.1016/j.eururo.2014.07.033 25151017

[42] Tyagi A Wu S-Y Sharma S Wu K Zhao D Deshpande R. Exosomal miR-4466 from nicotine-activated neutrophils promotes tumor cell stemness and metabolism in lung cancer metastasis. Oncogene. (2022) 41:3079–92. doi: 10.1038/s41388-022-02322-w PMC9135627 35461327

[43] Qu K Gu J Ye Y Williams SB Dinney CP Wu X. High baseline levels of interleukin-8 in leukocytes and urine predict tumor recurrence in non-muscle invasive bladder cancer patients receiving bacillus Calmette-Guerin therapy: A long-term survival analysis. Oncoimmunology. (2017) 6:e1265719. doi: 10.1080/2162402X.2016.1265719 28344874 PMC5353920

