# Supplementary material for: Development and external validation of a model to predict recurrence in patients with non-muscle invasive bladder cancer
Source: Front Immunol. 2025 Jan 10;15:1467527. doi: 10.3389/fimmu.2024.1467527 (PMC11757240; doi:10.3389/fimmu.2024.1467527)
Supplement: Supplementary file 1 [file Table1.docx]

Supplementary Material

# 1 Supplementary Table 1

The relationship between postoperative recurrence and clinicopathological parameters of NMIBC.

| **Variables** | **Total（%）** | **Recurrence（n=249）** | **Non- recurrence（n=307)** |
| --- | --- | --- | --- |
| Age |  |  |  |
| ＜60 | 198（35.6） | 68（12.2） | 130（23.4） |
| ≥60 | 358（64.4） | 181（32.6） | 177（31.8） |
| Gender |  |  |  |
| Male | 446（80.2） | 199（35.8） | 247（44.4） |
| Female | 110（19.8） | 50（9.0） | 60（10.8） |
| Blood type |  |  |  |
| A | 200（36.0） | 91（16.4） | 109（19.6） |
| B | 142（25.5） | 63（11.3） | 79（14.2） |
| AB | 57（10.3） | 20（3.6） | 37（6.7） |
| O | 157（28.2） | 75（13.5） | 82（14.7） |
| Smoking |  |  |  |
| Yes | 154（27.7） | 71（12.8） | 83（14.9） |
| No | 402（72.3） | 178（32.0） | 224（40.3） |
| Diabetes mellitus |  |  |  |
| Yes | 50（9.0） | 24（4.3） | 26（4.7） |
| No | 506（91.0） | 225（40.5） | 281（50.5） |
| Hypertension |  |  |  |
| Yes | 143（25.7） | 69（12.4） | 74（13.3） |
| No | 413（74.3） | 180（32.4） | 233（41.9） |
| Cerebrovascular disease |  |  |  |
| Yes | 17（3.1） | 8（1.5） | 9（1.6） |
| No | 539（96.9） | 241（43.3） | 298（53.6） |
| Urinary system disease |  |  |  |
| Yes | 235（42.3） | 112（20.1） | 123（22.1） |
| No | 321（57.7） | 137（24.6） | 184（33.1） |
| Tumor number |  |  |  |
| Single | 248（44.6） | 45（8.1） | 203（36.5） |
| Multiple | 308（55.4） | 204（36.7） | 104（18.7） |
| Tumor size |  |  |  |
| ≤3cm | 453（81.5） | 199（35.8） | 254（45.7） |
| ＞3cm | 103（18.5） | 50（9.0） | 53（9.5） |
| Tumor location |  |  |  |
| Side wall | 392（70.5） | 188（33.8） | 204（36.7） |
| Bladder neck | 54（9.7） | 20（3.6） | 34（6.1） |
| Trigone | 51（9.2） | 22（4.0） | 29（5.2） |
| Other | 59（10.6） | 19（3.4） | 40（5.2） |
| Growth pattern |  |  |  |
| Papilla | 115（20.7） | 48（8.6） | 67（12.1） |
| Cauliflower | 149（26.8） | 70（12.6） | 79（14.2） |
| Coralloid | 59（10.6） | 30（5.4） | 29（5.2） |
| Carpet | 18（3.2） | 7（1.3） | 11（2.0） |
| Waterweed | 82（14.7） | 37（6.7） | 45（8.1） |
| Other | 133（23.9） | 57（10.3） | 76（13.7） |
| Infusion medication |  |  |  |
| HydroxycamptotHecin | 380（68.3） | 179（32.2） | 201（36.2） |
| Pirarubicin | 176（31.7） | 70（12.6） | 106（19.1） |
| Tumor stage |  |  |  |
| Ta | 230（41.4） | 105（18.9） | 125（22.5） |
| T1 | 326（58.6） | 144（25.9） | 182（32.7） |
| Tumor grade |  |  |  |
| Low malignant potential | 166（29.9） | 67（12.1） | 99（17.8） |
| Low | 286（51.4） | 126（22.7） | 160（28.8） |
| High | 104（18.7） | 56（10.1） | 48（8.6） |
| U-LEU |  |  |  |
| Positive | 223（41.1） | 167（30.0） | 56（10.1） |
| Negative | 333（59.9） | 82（14.7） | 251（45.1） |
| BLD |  |  |  |
| Positive | 114（20.5） | 110（19.8） | 4（0.7） |
| Negative | 442（79.5） | 139（25.0） | 303（54.5） |
| Urinary protein |  |  |  |
| Positive | 19（3.4） | 13（2.3） | 6（1.1） |
| Negative | 537（96.6） | 236（42.2） | 301（54.1） |
| Urine nitrite |  |  |  |
| Positive | 65（11.7） | 33（5.9） | 32（5.8） |
| Negative | 491（88.3） | 216（38.8） | 275（49.5） |
| Blood sugar | - | 4.9（4.4~5.9） | 4.78（4.45~5.3） |
| Creatinine | - | 82（73~98） | 80（70~91） |
| Uric acid | - | 342（282~406） | 338（279~391） |
| Total protein | - | 64.6（61.1~69.4） | 65.2（60.9~68.9） |
| Albumin | - | 39.0±4.0 | 39.7±3.7 |
| Globulin | - | 26（23~29） | 25（22.4~27.9） |
| A/G | - | 1.5（1.3~1.7） | 1.6（1.4~1.8） |
| Platelet | - | 159（113~192） | 193（163~235） |
| Leukocyte | - | 6.0（4.8~7.4） | 5.8（4.9~6.9） |
| Erythrocyte | - | 4.4（3.9~4.7） | 4.4（4.1~4.8） |
| Hemoglobin | - | 133.0（119.5~144.0） | 137（123~147） |
| Thrombin time | - | 19.4（16.9~21.5） | 17.9（17.0~19.6） |
| Fibrinogen | - | 2.8（2.7~3.3） | 2.7（2.3~3.3） |
| Red blood cell distribution width | - | 13.3（12.8~14.1） | 13.1（12.5~13.7） |
| U-LEU | - | 8.0（0.0~40.5） | 5.0（0.0~23.0） |
| Activated partial Prothrombin | - | 29.2（25.9~34.2） | 27.1（25.2~31.7） |
| Alanine aminotransferase | - | 17（13~25） | 24.0（20.0~28.0） |
| Aspartate aminotransferase | - | 24（19~29） | 18.0（13.8~26.3） |
| AST/ALT | - | 1.3（1.0~1.7） | 1.3（1.0~1.6） |

# 2 Supplementary Table 2

Evaluation of the Prediction Performance of the Random Survival Forest Model

| **index** | **training set** | **validation set** |
| --- | --- | --- |
| Specificity | 0.915 | 0.800 |
| Sensibility | 0.909 | 0.826 |
| Accuracy | 0.911 | 0.817 |
| Precision | 0.927 | 0.882 |
| Recal | 0.909 | 0.826 |
| F1 value | 0.918 | 0.853 |
